# Avoiding Unnecessary Major Rectal Cancer Surgery by Implementing Structural Restaging and a Watch-and-Wait Strategy After Neoadjuvant Radiochemotherapy

**DOI:** 10.1245/s10434-020-09192-0

**Published:** 2020-11-10

**Authors:** J. F. Huisman, I. J. H. Schoenaker, R. M. Brohet, O. Reerink, H. van der Sluis, F. C. P. Moll, E. de Boer, J. C. de Graaf, W. H. de Vos tot Nederveen Cappel, G. L. Beets, H. L. van Westreenen

**Affiliations:** 1 Department of Gastroenterology and Hepatology, Isala Hospital, Zwolle, The Netherlands; 2 Department of Surgery, Isala Hospital, Zwolle, The Netherlands; 3 Department of Epidemiology and Statistics, Isala Hospital, Zwolle, The Netherlands; 4 Department of Radiotherapy, Isala Hospital, Zwolle, The Netherlands; 5 Department of Pathology, Isala Hospital, Zwolle, The Netherlands; 6 Department of Radiology, Isala Hospital, Zwolle, The Netherlands; 7 Department of Medical Oncology, Isala Hospital, Zwolle, The Netherlands; 8 Department of Surgery, The Netherlands Cancer Institute, Amsterdam, The Netherlands; 9 GROW School for Oncology and Developmental Biology, Maastricht University, Amsterdam, The Netherlands

## Abstract

**Background:**

Pathologic complete response (pCR) after neoadjuvant chemoradiotherapy (nCRT) is found in 15–20% of patients with locally advanced rectal cancer. A watch-and-wait (W&W) strategy has been introduced as an alternative strategy to avoid surgery for selected patients with a clinical complete response at multidisciplinary response evaluation. The primary aim of this study was to evaluate the efficacy of the multidisciplinary response evaluation by comparing the proportion of patients with pCR since the introduction of the structural response evaluation with the period before response evaluation.

**Methods:**

This retrospective cohort study enrolled patients with locally advanced rectal cancer who underwent nCRT between January 2009 and May 2018, categorizing them into cohort A (period 2009–2015) and cohort B (period 2015–2018). The patients in cohort B underwent structural multidisciplinary response evaluation with the option of the W&W strategy. Proportion of pCR (ypT0N0), time-to-event (pCR) analysis, and stoma-free survival were evaluated in both cohorts.

**Results:**

Of the 259 patients in the study, 21 (18.4%) in cohort A and in 8 (8.7%) in cohort B had pCR (*p* = 0.043). Time-to-event analysis demonstrated a significant pCR decline in cohort B (*p* < 0.001). The stoma-free patient rate was 24% higher in cohort B (*p* < 0.001).

**Conclusion:**

Multidisciplinary clinical response evaluation after nCRT for locally advanced rectal cancer led to a significant decrease in unnecessary surgery for the patients with a complete response.

The standard therapy for locally advanced rectal cancer is neoadjuvant chemoradiotherapy (nCRT) to downstage the tumor followed by surgical resection according to the principles of total mesorectal excision (TME). Despite a favorable oncologic outcome, TME is accompanied with perioperative mortality and morbidity.^1^ Histopathology of resected specimens shows disappearance of malignant tumor and lymph nodes—a pathologic complete response (pCR)—in 15–20% of patients.^2^ In 2004, Habr-Gama et al.^3^ proposed a watch-and-wait (W&W) policy rather than TME surgery for patients with an apparent clinical complete response (cCR). Since then, several other studies have reported on the clinical outcome and oncologic safety of the W&W policy.^4^^,^^5^ A recent study even advocated an extended observation period for patients with a near cCR.^6^ In the last decade, interest in these organ-preservation strategies for selected patients has been increasing, with the aim to improve the quality of life for cancer survivors.

Organ preservation starts with a structural multidisciplinary response evaluation after CRT to identify the patients with a good response. The primary aim of our study was to evaluate the efficacy of this multidisciplinary response evaluation for locally advanced rectal cancer by comparing the number of pCRs (unnecessary surgeries) after nCRT for patients who had no multidisciplinary response evaluation with that for patients who underwent structural multidisciplinary response evaluation in our colorectal unit.

## Methods

### Patient Inclusion and Selection

This retrospective cohort study was performed in a Dutch high-volume colorectal cancer center. All patients identified with locally advanced rectal cancer who underwent a long-course nCRT with curative intent (28 fractions of 1.8-Gy radiotherapy with a twice daily bolus of capecitabine 825 mg/m^2^) between January 2009 and June 2018 were enrolled in the study and assigned to cohort A or B.

**Fig. 1:**
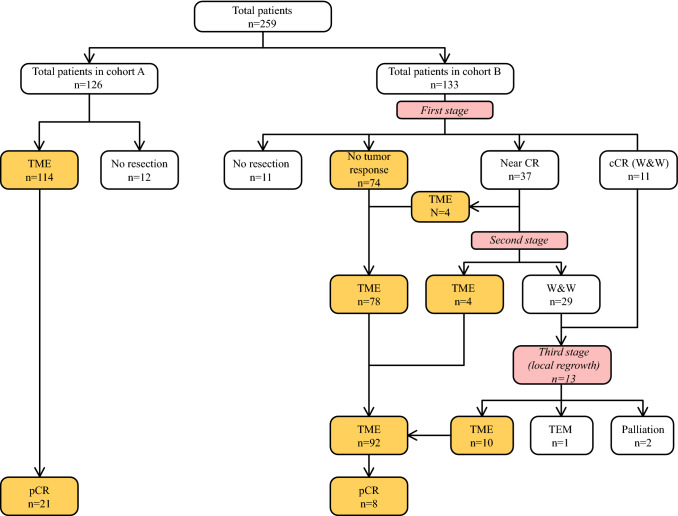
Three stage treatment algorithm for systematic evaluation for TME in cohort A & B. First stage was first multidisciplinary discussion after nCRT. Second stage was second multidisciplinary discussion for patients with near cCR. Third stage was local regrowth W&W group. cCR = clinical Complete Response. W&W = Watch and Wait. TME = Total Mesorectal Excision. TEM = Transanal Endoscopic Microsurgery

Cohort A consisted of patients without local response evaluation after nCRT (period 2009–2015) who received either a TME resection or further palliative treatment due to the development of widespread distant metastases or a poor condition (Fig. 1). Cohort B consisted of patients who had response evaluation after nCRT (period 2015–2018) with digital rectal examination, diffusion-weighted (DWI) magnetic resonance imaging (MRI), sigmoidoscopy, and computed tomography (CT).

The patients in cohort B were categorized as having cCR, near cCR, or obvious residual tumor or palliation after nCRT (Fig. 1). The patients with obvious residual tumor underwent a TME resection, and the patients with widespread metastases or a poor condition underwent palliative treatment. The patients with a cCR or near cCR on response evaluation entered the W&W program, as described later. All imaging methods for the patients with cCR or near cCR were referred to the Antoni van Leeuwenhoek hospital (AVL) in Amsterdam, an expert center for W&W, to have a second reading before inclusion in the W&W group.

The patients were categorized as having “cCR” when both endoscopic and radiologic cCRs were achieved or as having “near cCR” if endoscopic or radiologic near cCR was achieved. Endoscopic cCR was defined as a white scar with or without telangiectasia and no palpable abnormalities. Radiologic cCR was defined as the absence of residual tumor on T2W-MRI, with a low signal at the former tumor location on b1000 DWI-MRI and the absence of suspicious lymph nodes on T2W-MRI. Endoscopic near-complete response was defined as a superficial soft irregularity on digital rectal examination, a small residual flat ulcer, or irregular wall-thickening at endoscopy and/or adenomatous tissue with dysplasia at histopathologic examination. Radiologic near cCR was defined as obvious downstaging with or without residual fibrosis but with a heterogeneous or irregular aspect on MRI and/or a small focal area of high signal on b1000 DWI-MRI.^6^ The patients with involved mesorectal fascia (MRF) or involved local organs after nCRT were referred to a tertiary center for TME with intraoperative radiotherapy (IORT). The study was approved by the medical ethics committee (reference no. 180805).

### Follow-up Surveillance Procedure for cCR and Near cCR After nCRT

The treatment and follow up decisions can be regarded as a three-stage treatment stratification algorithm over time (Fig. 1). In general, the first response evaluation was planned to occur 8 weeks after completion of the nCRT, with the second response evaluation planned to occur 12–16 weeks after the first response evaluation.

After the first-stage response evaluation, the patients with cCR after nCRT were offered the W&W policy and underwent intensive follow-up evaluation with endoscopy, rectal MRI, abdominal and thoracic CT, and carcinoembryonic antigen (CEA) screening every 3–6 months (Table 1). The patients with a near cCR at the first-stage response evaluation were offered TME or second-stage response evaluation.

**Table 1:** Follow-up watch and wait

Year	1				2				3		4		5	
Months	3	6	9	12	15	18	21	24	30	36	42	48	54	60
CEA	x	x	x	x	x	x	x	x	x	x	x	x	x	x
Endoscopy	x	x	x	x		x		x	x	x	x	x	x	x
Rectal MRI	x	x		x		x		x	x	x	x	x	x	x
Thoracic and abdominal CT		x		x		x		x		x		x		x

x means that the diagnostic test was scheduled

*CEA* carcinoembryonic antigen; *MRI* magnetic resonance imaging; *CT* computed tomography

A second-stage response evaluation was performed for the patients with near cCR who did not choose to undergo TME. At this evaluation, the patients were classified as cCR or no cCR. The patients without cCR underwent TME, whereas the patients with cCR were offered the W&W policy with intensive follow up evaluation.

In the third stage (follow-up W&W), the patients submitted for W&W who showed local regrowth at any time during the follow-up period were considered for salvage TME or local excision. The patients with incurable distant metastasis were offered palliative therapy.

### Statistical Analysis

The primary outcome of the study was the proportion of pCR defined as the absence of malignant tumor and lymph nodes in the pathologic TME resection specimen (ypT0N0). The secondary outcomes were the stoma-free patient rate and the disease-free survival (DFS) rate in both cohorts. All analyses were performed using Statistical Package of Social Sciences version 24.0 (SPSS, Armonk, NY). A *p* value lower than 0.05 was considered significant. Descriptive statistics were reported as median with range or as count with proportion.

The baseline characteristics of cohorts A and B were compared using the Mann–Whitney *U* test for continuous variables and the Chi square test for categorical variables. Univariate analysis was performed to determine the difference between the patient characteristics of cohorts A and B at baseline. Kaplan–Meier survival analysis was used to estimate the probability of pCR after nCRT, local regrowth in the W&W group, and DFS.

For survival analyses of pCR and local regrowth, the start of the follow-up evaluation was the date of the last nCRT, and the end of the follow-up evaluation was the date of interest. For the analysis of pCR, the end of the follow-up evaluation was the date of the TME resection, the date of the last follow-up scan, the date of tumor progression, or the date of death or the date of the decision not to perform TME (palliative group), whichever came first. The patients who underwent TME after the second response evaluation or salvage TME due to local regrowth during W&W were counted in the TME group (Fig. 1). For the analysis of local regrowth, the end of the follow-up evaluation was the date of local regrowth or the date of the last follow-up endoscopy/MRI.

A DFS analysis (non-endoluminal or distant recurrence) was performed for the patients who underwent curative surgery without distant metastasis after nCRT and for the patients submitted to W&W. The start of the follow-up evaluation was the date of curative surgery or the decision for W&W. The end of the follow-up evaluation was the date of recurrence, the date of death, or the date of the last CT, whichever came first. For the stoma-free analysis, we calculated the presence of a stoma at the end of the follow-up period using proportions.

## Results

### Baseline Characteristics

The study enrolled 259 patients: 126 patients in cohort A and 133 patients in cohort B. The baseline characteristics, presented in Table 2, differed significantly between the two cohorts in terms of clinical T stage and N stage.

**Table 2:** Baseline characteristics

	Cohort A	Cohort B	*p* value
	*n* (%)	*n* (%)	
*n*	126	133	
Male**s**	71 (56)	87 (65)	0.135^a^
Median age: years (range)	66 (32–85)	65 (34–88)	0.354^b^
Median tumor high from AV: cm (range)	6 (2–15)	6 (0–17)	0.883^b^
*cT-stage*			0.027^a^
T2	2 (2)	7 (5)	
T3	104 (83)	94 (71)	
T4	15 (12)	25 (19)	
T3/4	1 (1)	6 (5)	
Missing	4	1	
cN+	109 (95)	114 (86)	< 0.001^a^
cN0	6	18	
cNx	11	1	

*AV* anal verge

^a^Chi square test

^b^Mann-Whitney *U* test

### Cohort A

In cohort A, 114 patients (90%) underwent TME. For the remaining 12 patients, TME was not performed due to the development of incurable distant metastasis after nCRT (*n* = 6) or comorbidity (*n *= 3), or because of patient preference (*n* = 1), and two patients died of sepsis during nCRT.

### Cohort B

For cohort B, the third-stage treatment algorithm for stratification over time was evaluated (Fig. 1).

#### First-Stage Response Evaluation

All the patients in cohort B underwent first-stage multidisciplinary response evaluation. The median observational interval between the end of nCRT and the response evaluation was 8 weeks (range, 5–22 weeks). At this response evaluation, 11 patients (8%) had cCR, 37 patients (28%) had near cCR, 74 patients (56%) had obvious residual tumor, and 11 patients (8%) were assigned to the palliative group (Fig. 1; Table 3).

**Table 3:** Treatment stratification at first response evaluation in cohort B

	Cohort A	Cohort B
	*n* (%)	*n* (%)
No. of patients	126	133
Primary TME	114 (90)	74 (56)
No resection	12 (10)	11 (8)
cCR (W&W)	–	11 (8)
Near cCR	–	37 (28)

*TME* total mesorectal excision; *cCR* clinical complete response; *W&W* watch and wait

All 74 patients with obvious residual tumor underwent TME. For 11 patients, palliative treatment was administered because of distant metastasis (*n* = 5), comorbidity (*n* = 3), or patient preference (*n* = 1), and two patients died due to bowel perforation and cardiovascular event.

When 11 patients showed a cCR after the first response evaluation on both endoscopy and MRI, they were entered into the W&W surveillance program. Near cCR was observed in 37 patients (28%), with 15 patients (41%) showing possible residual tumor on both endoscopy and MRI, 15 patients (41%) showing possible residual tumor on endoscopy and cCR on MRI, and 7 patients (19%) showing possible residual tumor on MRI and cCR on endoscopy. Of these 37 patients, 4 underwent primary TME instead of second-response evaluation due to patient preference (*n* = 3) or symptomatic rectal stenosis (*n* = 1). The remaining 33 patients underwent second-stage response evaluation.

#### Second-Stage Response Evaluation

Second-stage response evaluation was performed for the 33 patients (25%) with near cCR after a median of 13 weeks (range, 4–26 weeks) from the first response evaluation. After this response evaluation, 29 patients (88%) were submitted to the W&W. Four patients (12%) had obvious residual tumor and underwent TME. All operations were radical resections with free resection margins (R0), with histopathology showing ypT3N0 (*n* = 2) and ypT2N0 (*n* = 2) (Fig. 1).

#### Third-Stage Response or W&W Evaluation

Altogether, 40 patients were submitted to the definitive W&W surveillance program (11 patients after first-stage and 29 patients after second-stage response evaluation) (Fig. 1). Local regrowth occurred for 13 patients (32.5%) after a median of 14 months (range, 4–29 months). Of these 13 patients, 11 underwent successful curative and radical salvage surgery (R0) (TME for 10 patients and transanal endoscopic microsurgery [TEM] for 1 patient), and 1 refused TME, with the remaining patient undergoing palliative therapy for incurable osseous metastasis that developed 9 months after nCRT. Local or distant recurrence after salvage TME did not occur in our population.

**Fig. 2:**
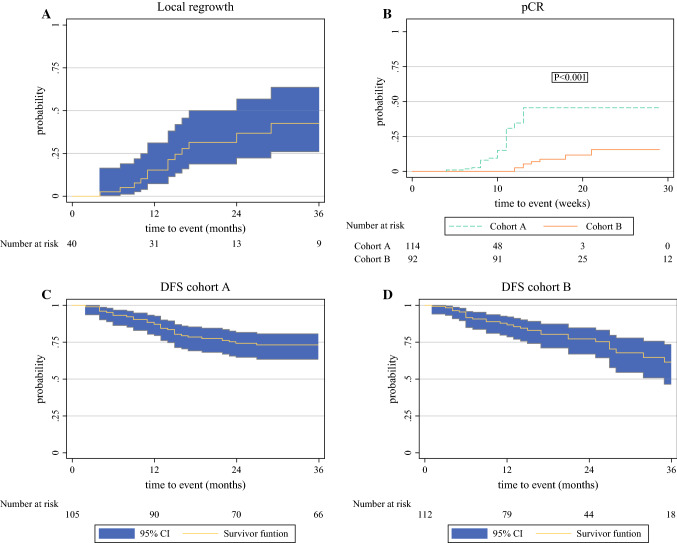
**(A)**: 3-year cumulative incidence of local regrowth among the W&W cohort (n=40). **(B)**: 30-week cumulative incidence of pCR after nCRT (n=206). **(C)**: 3-year disease free survival (DFS) among patients with curative therapy after nCRT in cohort A. **(D)** 3-year disease free survival (DFS) among patients with curative therapy after nCRT in cohort B

The 3-year cumulative incidence of local regrowth after nCRT among the W&W patients was 42% (95% confidence interval [CI], 26–64%; Fig. 2A). Two patients were censored during the W&W follow-up period for incurable distant metastasis (without local regrowth) after 5 and 23 months, respectively.

### Comparison of TME Outcome

Of the 259 patients, 206 (79.5%) underwent TME (114 patients in cohort A and 92 patients in cohort B). Two patients in cohort A and eight patients in cohort B underwent TME with IORT. The overall R0 resection rate was 95% in both cohorts. The findings showed pCR for 21 patients (16.7%) in cohort A and 8 patients (6%) in cohort B (*p* = 0.006). Among the patients who underwent TME (*n* = 206), the proportion of pCR was 18.4% in cohort A and 8.7% in cohort B (*p *= 0.043) (Table 4). The overall interval between nCRT and TME differed significantly. For the overall TME group (*n* = 206), the interval was 9 weeks (range, 4–29 weeks) in cohort A and 15 weeks (range, 9–129 weeks) in cohort B (*p* < 0.001), and for the patients with pCR (*n* = 29), the interval was 10 weeks (range, 4–13 weeks) in cohort A and 14 weeks (range, 12–21 weeks) in cohort B (*p* < 0.001). This indicates that the variable period between nCRT and TME may have affected the outcome. Therefore, we performed a survival analysis to account for this variable period over time. The 30-week cumulative incidence of pCR was 46% (95% CI, 30–65%) for the patients without a multidisciplinary response evaluation (cohort A) and 16% (95% CI, 7–31%) for the patients with a multidisciplinary response evaluation (cohort B) (P_log-rank_ < 0.001; Fig. 2B).

**Table 4:** Clinical outcome (pCR)

	Cohort A	Cohort B	*p* value
	*n* (%)	*n* (%)	
Median interval between nCRT and TME: weeks (range)	9 (4–29)	15 (9–129)	< 0.001^a^
pCR in total cohort (*n* = 259)	126	133	
pCR	21 (16.7)	8 (6)	0.006^b^
No pCR	104 (82.5)	125 (94)	
Missing	1	0	
pCR for patient with TME (*n* = 206)	114	92	
pCR	21 (18.4)	8 (8.7)	0.043^b^
No pCR	93 (80.7)	84 (91.3)	
Missing	1	0	
30-Week cumulative probability of pCR: % (95% CI)	46 (30–65)	16 (7–31)	< 0.001^c^

*pCR* pathologic complete response; *nCRT* neoadjuvant chemoradiotherapy; *TME* total mesorectal excision; *CI* confidence interval

^a^Mann–Whitney *U* test

^b^Chi square test

^c^Log-rank test

### Disease-Free Survival

The DFS analysis included 225 patients (109 in cohort A and 116 in cohort B) who underwent curative surgery or were submitted to W&W. For eight of these patients, follow-up evaluation was not performed or will be performed in future in case of a recent diagnosis. Local or distant recurrence occurred for 27 patients (24.8%) in cohort A and 27 patients (23.3%) in cohort B (Table 5). The 3-year DFS rate was 73% (95% CI, 63–81%) in cohort A and 61% (95% CI, 46–73%) in cohort B. (Figure 2C and D).

**Table 5:** Distant and local recurrence

	Cohort A	Cohort B	*p* value
	*n* (%)	*n* (%)	
Total cohort	126	133	
Patients included for DFS analysis	109	116	
Total recurrence	27 (24.8)	27 (23.3)	0.793^a^
Distant recurrence	23 (21.1)	21 (18.1)	
Local recurrence after TME	1 (0.9)	4 (3.4)	
Local and distant recurrence after TME	3 (2.8)	2 (1.7)	
Median time until recurrence: months (range)	13 (2–27)	12 (1–35)	0.768^b^
No recurrence	82 (75)	89 (76.7)	0.793^a^
Median follow-up: months (range)	58 (0–97)	20 (0–49)	< 0.001^b^
3-Year disease-free survival: % (95% CI)	73 (63–81)	61 (46–73)	

*DFS* disease-free survival; *TME* total mesorectal excision; *CI* confidence interval

^a^Chi square test

^b^Mann–Whitney *U* test

^c^Log-rank test

### Stoma-Free Survival

A stoma was created for 106 patients (84%) in cohort A and 84 patients (63%) in cohort B. Stoma reversal during the follow-up evaluation was performed for 23 patients in cohort A and 29 patients in cohort B. The median time to stoma reversal was 4 months (range, 0–9 months) in cohort A and 3 months (range, 0–33 months) in cohort B. At the end of the follow-up period, 43 patients (34%) in cohort A and 77 patients (58%) in cohort B were stoma-free (*p* < 0.001).

## Discussion

This study demonstrated a significant decline in TME resections without residual tumor (pCR) since the implementation of the structural multidisciplinary response evaluation after nCRT for locally advanced rectal cancer with the option of a W&W policy for patients with a very good response. With our current approach, the proportion of pCRs after TME decreased from 18 to 9%, and overall, a major TME resection could be avoided for more than 20% of patients, resulting in a 24% increase in stoma-free patients.

In our unit, the goal of structural response evaluation after nCRT is to identify patients with a very good response and offer them the option of organ preservation. To identify as many complete responders as possible, we allow patients with a near cCR at the first-stage response evaluation to have a longer observation period. The idea of this extended period is to maximize the detection of complete responders because current diagnostic techniques are not sufficiently accurate to detect true complete responders, accepting a higher local regrowth rate. A recent study reported that a longer observational period is safe and has no impact on oncologic outcome.^6^ With this policy, 40 patients (30%) entered the W&W surveillance program, and 92 patients (69%) underwent TME. Even with our liberal policy of a longer observation period for near complete responders, we had eight patients (6%) who showed a pCR after a TME. Two of these patients had a symptomatic rectal stenosis, and six of the patients had residual abnormalities at endoscopy. A recent paper showed that the majority of missed complete responses were due to residual abnormalities at endoscopy, and to a lesser extent, suspicious findings on MRI such as a high signal on T2W images, diffusion restriction, or dubious lymph nodes.^7^ The 3-year cumulative incidence of local regrowth among the W&W group was 42%, which is higher than the 2-year cumulative incidence of 25% in a recent registry study.^5^ This recent study also included patients with early rectal cancer (cT1-T2), whereas our study included almost exclusively cT3-4 tumors. Findings have shown an association between a higher original T stage in a W&W program and a higher regrowth rate.^8^ Additionally, we also included patients with a near cCR, and it is suggested that these patients also have a higher regrowth rate.^6^ Local regrowth seems to be associated with a higher incidence of distant recurrence.^5^, ^9^ In our study, only 1 (2.5%) of the 40 patients experienced local regrowth with distant metastasis during the W&W. The remaining patients were or could have been treated curatively with salvage surgery, indicating that structural response evaluation with the possibility of the W&W policy might be oncologically safe. Future research should investigate the exact timing and strategy of the restaging and factors associated with cCR or near cCR to maximize the detection of cCR and minimize the local regrowth rate.

The strength of our study was the comparison of pCR before and after the introduction of structural response evaluation for all patients with locally advanced rectal cancer who underwent nCRT, whereas most studies have focused only on the outcome of W&W. For this analysis, we used complete follow-up information on patients who had cCR, near cCR, or obvious residual tumor after response evaluation, and we also reported a complete follow-up evaluation of the patients before multidisciplinary response evaluation was introduced.

A limitation of this study was that the interval between nCRT and TME surgery differed between the two cohorts. Although the first multidisciplinary response evaluation was performed after 8 weeks in both cohorts, the prolonged interval between CRT and TME among the W&W patients might have influenced the tumor response.

Another limitation was that the study was performed in a single center with a relatively small number of patients, making it difficult to extrapolate the results to other settings and to perform accurate survival analysis. The study was underpowered to perform adequate DFS analysis. Furthermore, the difference in the median follow-up period between the two cohorts made it difficult to perform accurate DFS analysis.

Selection bias might have been introduced by historical influences over the years, such as the introduction of (re)staging MRI, an nCRT indication for patients with N1, and a prolonged observational interval between nCRT and restaging. Furthermore, the definition of near cCR is relatively subjective.

In conclusion, we reported a significant decrease in unnecessary surgery for patients with a complete response since the implementation of structural response evaluation and a W&W program for patients with locally advanced rectal cancer.
